# Correspondence between salivary proteomic pattern and clinical course in primary Sjögren syndrome and non-Hodgkin's lymphoma: a case report

**DOI:** 10.1186/1479-5876-9-188

**Published:** 2011-11-02

**Authors:** Chiara Baldini, Laura Giusti, Federica Ciregia, Ylenia Da Valle, Camillo Giacomelli, Elena Donadio, Francesco Ferro, Sara Galimberti, Valentina Donati, Laura Bazzichi, Stefano Bombardieri, Antonio Lucacchini

**Affiliations:** 1 Department of Internal Medicine, Rheumatology Unit, University of Pisa, Pisa, Italy; 2 Department of Psychiatry, Neurobiology, Pharmacology and Biotechnology, University of Pisa, Pisa, Italy; 3 Department of Oncology, Transplants and New Medical Techniques, Hematology Unit, University of Pisa, Pisa, Italy; 4 Pathological Anatomy Hospital Unit, Pisa, Italy

**Keywords:** primary Sjögren's Syndrome, B-cell non-Hodgkin's lymphoma, proteomic analysis

## Abstract

**Background:**

In the last years human proteomic has represented a promising tool to promote the communication between basic and clinical science.

**Methods:**

To explore the correspondence between salivary proteomic profile and clinical response, herein, we used a proteomic approach to analyse the whole saliva of a patient with primary Sjögren's Syndrome (pSS) and non-Hodgkin's-MALT type parotid lymphoma before, during and after a standard treatment with cyclophosphamide (CTX) and rituximab (RTX). To identify any discriminatory therapeutic salivary biomarker patient's whole saliva was collected at the baseline, after the fourth infusion of rituximab, and on remission and analysed combining two-dimensional electrophoresis (2DE) and MALDI-TOF/TOF mass spectrometry.

**Results:**

Proteomic results obtained from the comparison of salivary samples indicated several qualitative and quantitative modifications in the salivary expression of putative albumin, immunoglobulin J chain, Ig kappa chain C region, alpha-1-antitrypsin, haptoglobin and Ig alpha-1 chain C region.

**Conclusion:**

This study suggests that clinical and functional changes of the salivary glands driven by autoimmune and lymphoproliferative processes might be reflected in patients' whole saliva proteins, shedding new light on the potential usefulness of salivary proteomic analysis in the identification of prognostic and therapeutic biomarkers for patients with pSS and non Hodgkin's lymphomas.

## Background

Primary Sjögren's syndrome (pSS) is a mild chronic systemic autoimmune disease characterised by a slow progression and low morbidity and mortality rates [1,2]. Nonetheless, despite a generally rather indolent course, patients with pSS are widely known to have an increased risk for developing B-cell non-Hodgkin's lymphoma (B-cell NHL) [3]. To date, many efforts have been made in order to identify early predictors of B-cell NHL in pSS, but it is still difficult to identify who among the patients with pSS will have a progression to lymphoma [4]. In the last few years, proteomic analysis has been increasingly applied to the study of whole saliva in pSS in order to identify novel diagnostic biomarkers for the disease [5-7]. Only few studies have been carried out on the modification of salivary proteomic profiles during and after pharmacological treatments [8]. In this study, we used a proteomic approach to identify salivary therapeutic biomarkers in a patient with pSS and B-cell NHL-MALT type of the salivary glands, successfully treated with cyclophosphamide (CTX) and rituximab (RTX). Proteomic analysis of the patient's whole saliva, performed before, during the treatment course, and after 6 months of pharmacological therapy, showed an intriguing correspondence between the patient's clinical improvement and the changes of her proteomic salivary profile, highlighting a potential application of proteomics in identifying therapeutic biomarkers and in monitoring the efficacy of pharmacological treatments.

## Methods

### Patient and materials

**Figure 1:**
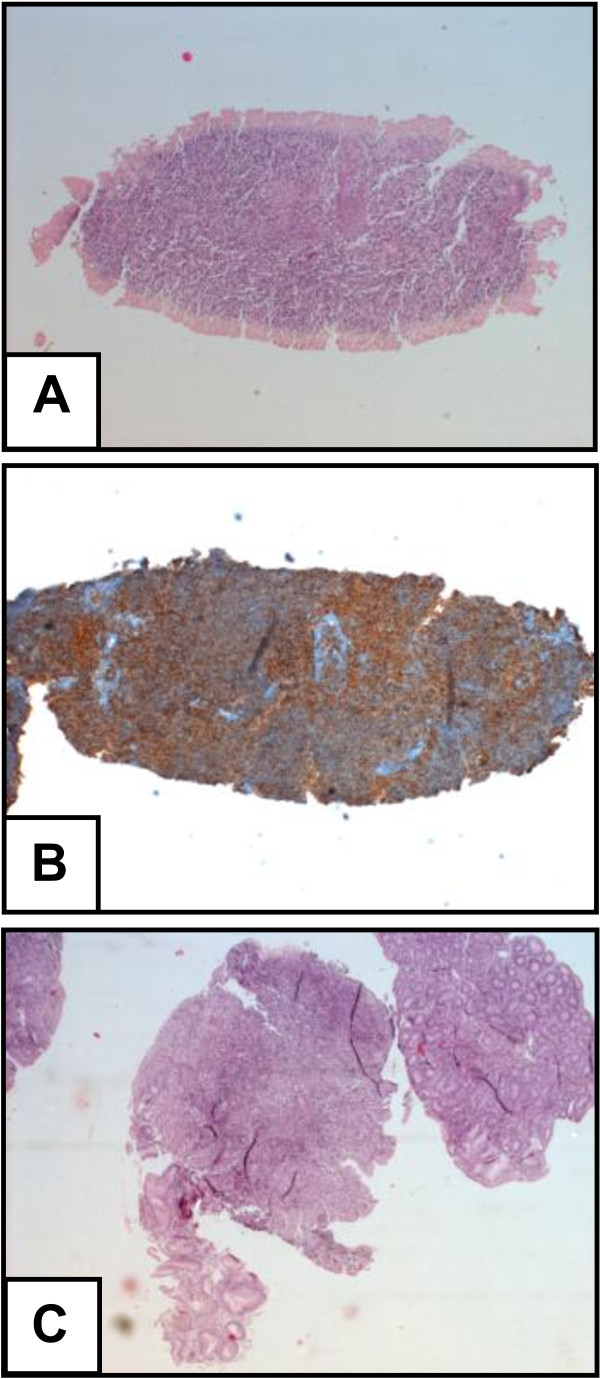
**Left parotid gland B cell non-Hodgkin's lymphoma (B-cell NHL), MALT type:** the infiltrate was focally positive for CD20 and bcl-2, the neoplastic cells were CD3, CD5, and cyclin D1 negative (A,B). Upper gastro-intestinal biopsy tissue: lymphoid infiltrate in lamina propria **(C)**.

Salivary samples were prospectively collected from a 53-year-old Caucasian woman presenting with a 6-year history of pSS and a novel diagnosis of parotid gland B cell-NHL, MALT type. The diagnosis of pSS has been made in 2003, according to the American and European consensus criteria (AECG) for the disease [9]. In 2009, she presented a persistent unilateral enlargement of the left parotid gland. A biopsy of the gland was performed leading to the diagnosis of parotid gland B cell-NHL, MALT type (Figure 1A and 1B). The patient was referred to our Unit for staging of lymphoma. At presentation, she denied fever, chills, night sweats, anorexia, and weight loss. Physical examination revealed a well-appearing woman with normal vital signs. The left parotid gland was markedly enlarged, but there were no signs of skin vasculitis or peripheral lymphadenopathy. Table 1 summarises the patient's blood sample results. A positron emission tomography scan showed only a focal area of mild tracer uptake in the left parotid gland (SUV 3.3). Bone marrow needle and biopsy showed a normal macroscopic appearance, and only IgH rearrangement was positive. Computed tomography of the chest, abdomen, and pelvis revealed no evidence of lymphadenopathy, enlarged spleen or liver involvement. Upper gastro-intestinal endoscopy (UGIE) showed a suspicious lymphoid infiltrate in lamina propria, probably reactive (Wotherspoon grade 4) (Figure 1C). The patient was then treated with the anti-CD20 monoclonal antibody rituximab (RTX) at 375 mg/m2, once a week, for four consecutive weeks, plus oral cyclophosphamide (CTX 100 mg/day for 15 days every month for four months) and went into complete remission by six months. Written informed consent was obtained from the patient for her inclusion in the study. The study was approved by local ethics committee "Comitato per la sperimentazione clinica dei farmaci, Azienda Ospedaliera Universitaria Pisana", reference number 3062.

**Table 1:** Patient's clinical and serological data

	Normal values	Baseline	During treatment (*)	On remission
**ESR**	< 30 mm	15 mm	14 mm	12 mm

**C reactive protein**	< 0.5 mg/dl	0.27 mg/dl	0.15 mg/dl	0.06 mg/dl

**IgG**	700 - 1600 mg/dl	1390 mg/dl	1290 mg/dl	1295 mg/dl

**IgM**	20 - 230 mg/dl	123 mg/dl	142 mg/dl	106 mg/dl

**Monoclonal component**	negative	negative	negative	negative

**White cells count**	4500 - 10000/mm^3^	5310/mm^3^	2610/mm^3^	3280/mm^3^

**Hemoglobin**	12 - 16 g/dl	14.2 g/dl	13.3 g/dl	13.7 g/dl

**Platelets**	150000 - 350000/mm^3^	211000/mm^3^	178000/mm^3^	170000/mm^3^

**Lactate dehydrogenase**	105 - 333 IU/L	213 IU/L	192 IU/L	194

**C4 levels**	10 - 40 mg/dl	8 mg/dl	12 mg/dl	11 mg/dl

**C3 levels**	90 - 180 mg/dl	81 mg/dl	86 mg/dl	89 mg/dl

**Antinuclear antibodies**	negative	positive speckled 1: 160	positive speckled 1: 160	positive speckled 1: 160

**Anti-Ro/SSA**	negative	positive	positive	positive

**Anti-La/SSB**	negative	negative	negative	negative

**Rheumatoid Factor**	< 15 IU/L	11 IU/L	9 IU/L	9 IU/L

**Cryoglobulinemia**	negative	negative	negative	negative

**CD 4**	36 - 50%	40.1%	43.2%	44.2%

**CD 8**	20 - 28%	38.6%	43.3%	39.4%

**CD 3**	65 - 77%	80.1%	89.3%	82.7%

**CD 19**	5 - 14%	7.8%	0.4%	0.8%

**CD 16+56**	3 - 15%	10.2%	7.7%	7.9%

(*) after the fourth infusion of rituximab

Unstimulated whole saliva specimens were collected early in the morning in standard conditions at baseline, after the fourth infusion of RTX, and after 6 months from the beginning of the treatment with CTX plus RTX regimen when clinical remission was obtained. Proteomic analysis was performed combining two dimensional electrophoresis (2DE) and MALDI-TOF/TOF mass spectrometry. Samples were treated as previously described [5]. Briefly, 150 μg of salivary proteins was loaded and the samples were applied by in-gel rehydration for 10 h using low voltage (30 V) in pH 3-10 L, 18 cm long IPG strips. The proteins were then focused for up to 70000 Vh at a maximum voltage of 8000 V. The second dimension (SDS-PAGE) was carried out by transferring the proteins to 12.5% polyacrylamide gel, running at 40 mA/gel and 10°C for about 6 h. Protein visualizations were obtained by silver staining [10], and a comparison of images was performed using Image Master 2D-platinum 6.01 (GE Health Care). The 2DE experiments were performed in triplicate. The significance of the differences (p-value < 0.05) was calculated using the Mann-Whitney test.

Spots of interest were cut out from the master gel and de-stained by washing with 50% ACN in 50 mM ammonium bicarbonate for 30 min. Gel pieces were then dried for 30 min in a Hetovac vacuum centrifuge (HETO, Allerod, Germany). Dried pieces of gel were subjected to protein digestion by trypsin and peptide extraction. MS and MS/MS analysis of peptides from 2-DE gel spots were performed with a 4800 Proteomics Analyzer MALDI-TOF/TOF mass spectrometer (Applied Biosystems, Framingham, MA, USA) according to the tuning procedures suggested by the manufacturer. Peak lists were generated with the Launch peak to MASCOT tools with the following settings: for the MS data, mass range 850-4000, peak density of maximum 20 peaks per 100 Da, minimal S/N ratio of 15, minimal area of 250, max peak 50; for the MS/MS data, mass range 60-2000; peak density of maximum 50 peaks per 200 Da, minimal S/N ratio of 5, minimal area of 20, and maximum number of peak set at 200. Such acquired MS and MS/MS data were compared to the database using MASCOT search engine http://www.matrixscience.com. In MASCOT, the combined PMF and MS/MS search was performed on uniprot_sptr_14.9-03-Mar-2009 database (selected for Homo sapiens, 87868 entries). Search settings allowed one missed cleavage with the trypsin enzyme selected, one fixed modification (carboxymethylated cysteine) and a variable modification (oxidation of methionine). Scaffold (version Scaffold_3_00_03, Proteome Software Inc., Portland, OR) was used to validate MS/MS based peptide and protein identifications. Peptide identifications were accepted if they could be established at greater than 95.0% probability as specified by the Peptide Prophet algorithm [11]. Protein identifications were accepted if they could be established at greater than 95.0% probability and contained at least 2 identified peptides. Protein probabilities were assigned by the Protein Prophet algorithm [12]. Proteins that contained similar peptides and could not be differentiated based on MS/MS analysis alone were grouped to satisfy the principles of parsimony [11,12].

## Results

**Figure 2:**
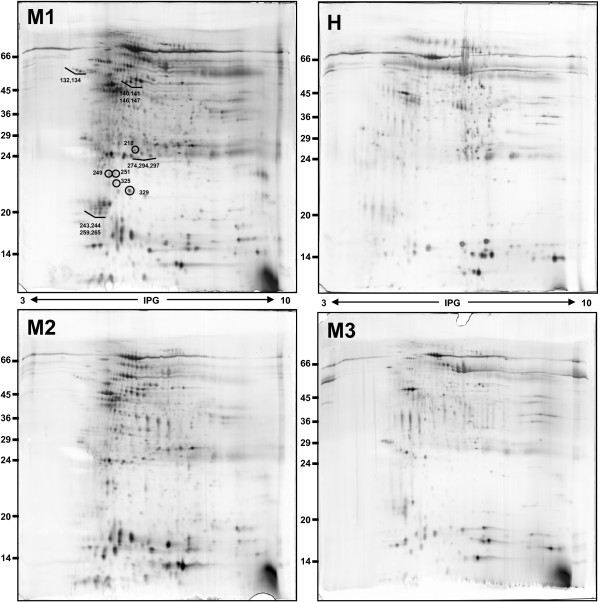
**Salivary proteome analysis**. Representative patient's salivary profiles at baseline **(M1)**, after the fourth infusion of RTX **(M2) **and on remission **(M3)**. Representative salivary pattern of a healthy subject **(H)**.

**Figure 3:**
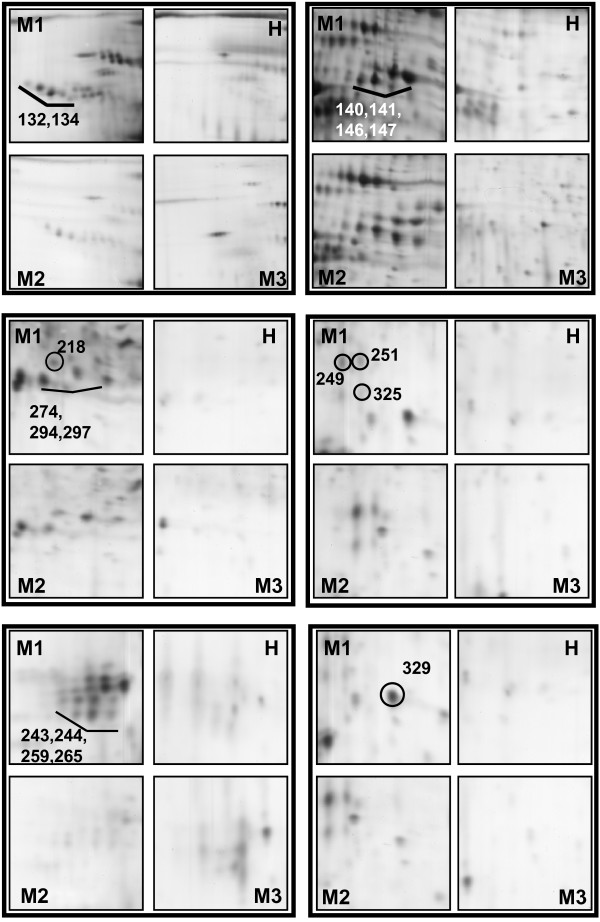
**Enlarged images of the 2D gels**. Enlarged images of the 2DE gels highlighting the differential expression of proteins identified at different conditions.

Figures 2 and 3 clearly illustrates the differences in the patient's salivary profile at baseline (M1), after the fourth infusion of RTX (M2) and after 6 months with the patient on remission (M3). Overall, 18 spots appeared to be differently expressed in the salivary profile of the patient at baseline and during treatment in comparison with the proteomic pattern obtained on remission. Mass spectrometry identification showed that these spots collapsed essentially on six main proteins, including putative uncharacterized albumin (spots n° 141, 146, 147, 249, 251, 274, 294, 297, 325), Immunoglobulin J chain (spots n°243, 244, 259, 265), Ig kappa chain C region (spot n° 218), alpha-1-antitrypsin (spots n°132, 134), Ig alpha-1 chain C region (spot n° 140) and haptoglobin (spot n° 329). A list of identified proteins, MW, pI, score and coverage values of MS/MS is shown in Table 2. From a qualitative point of view, when we compared the M1 and M3 profiles we observed a complete disappearance of many spots related to Ig alpha-1 chain C region, Ig kappa chain C region, Immunoglobulin J chain and putative uncharacterized protein albumin. From a quantitative point of view, the most relevant differences between M1 and M3 were detected for haptoglobin (5.8-fold decrease, p-value = 0.0019) and alpha-1-antitrypsin (6.3-fold decrease, p-value = 0.036). These two proteins were also characterised by a significant change in the expression level after the fourth infusion of RTX with haptoglobin and alpha-1-antitrypsin showing a 2.6-fold (p-value = 0.011) and a 2.1-fold (p-value = 0.05) decrease respectively, when compared to the baseline.

**Table 2:** Protein Identifications of differentially expressed proteins by Maldi TOF/TOF analysis

Spot n°	Protein name	Accession n°	MW/pI (theoretical)	Matched peptides	Coverage %	Best ion score	Peptides Identified	Fold variation M1 *vs *M3	p value
**132**	α-1-antitrypsin SERPINA1	P01009	46/5.37	3	9.3	95.7	(K)ITPNLAEFAFSLYR(Q)	6.3	0.036
**134**	α-1-antitrypsin SERPINA1	P01009	46/5.37	1	3.3	78.4	(K)ITPNLAEFAFSLYR(Q)	8.7	0.031
**140**	Ig α-1 chain C region	P01876	37/6.08	2	7.6	55.4	(R)WLQGSQELPR(E)	nd	nd
**141**	Putative uncharacterized protein ALB	A6NBZ8	71/6.33	11	24.1	85.6	(K)VFDEFKPLVEEPQNLIK(Q)	4	0.033
**146**	Putative uncharacterized protein ALB	A6NBZ8	71/6.33	7	16.4	78.3	(K)KVPQVSTPTLVEVSR(N)	11	0.012
**147**	Putative uncharacterized protein ALB	A6NBZ8	71/6.33	7	15	82.2	(K)DVFLGMFLYEYAR(R)	nd	nd
**249**	Putative uncharacterized protein ALB	A6NBZ8	71/6.33	5	11	74.7	(K)SLHTLFGDKLcTVATLR(E)	nd	nd
**251**	Putative uncharacterized protein ALB	A6NBZ8	71/6.33	5	10.5	91.2	(K)VHTEccHGDLLEcADDRADLAK(Y)	nd	nd
**274**	Putative uncharacterized protein ALB	A6NBZ8	71/6.33	2	2.5	49.8	(K)YLYEIAR(R)	nd	nd
**294**	Putative uncharacterized protein ALB	A6NBZ8	71/6.33	5	8.4	72.1	(R)FKDLGEENFK(A)	2	0.020
**297**	Putative uncharacterized protein ALB	A6NBZ8	71/6.33	5	9.1	61.7	(R)FKDLGEENFK(A)	nd	nd
**325**	Putative uncharacterized protein ALB	A6NBZ8	71/6.33	4	7	80.4	(K)SLHTLFGDKLcTVATLR(E)	nd	nd
**243**	Immunoglobulin J chain	P01591	15/5.12	5	33.6	111.3	(R)SSEDPNEDIVER(N)	2.1	0.016
**244**	Immunoglobulin J chain	P01591	15/5.12	5	33.6	112.4	(R)SSEDPNEDIVER(N)	3.6	0.005
**259**	Immunoglobulin J chain	P01591	15/5.12	3	16.8	75.2	(R)SSEDPNEDIVER(N)	4.1	0.006
**265**	Immunoglobulin J chain	P01591	15/5.12	4	22.6	69.8	(R)SSEDPNEDIVER(N)	2.8	0.025
**218**	Ig kappa chain C region	P01834	11/5.58	4	50	125.6	(K)SGTASVVcLLNNFYPR(E)	nd	nd
**329**	Haptoglobin	P00738	45/6.13	2	11.8	34.5	(K)AVGDKLPEcEADDGcPKPPEIAHGYVEHSVR(Y)	5.8	0.002

nd = not determined: corresponding to the % of volume of M3 spots was < 0.005

## Discussion

Recent advances in proteomic technologies have permitted the discovery of several salivary candidate biomarkers for the diagnosis of pSS [13-15]. So far, the potential usefulness of salivary proteomics for the discovery of therapeutic biomarkers has been scarcely investigated. Nonetheless, preliminary results have shown a modification of pSS proteomic profile before and after pilocarpine [8] This study, for the first time, demonstrated a strong correspondence between the salivary proteomic profile and the clinical disease course in a patient with pSS and B-cell NHL-MALT type of the salivary glands. In particular, at the baseline, we described the over-expression of seven different spots collapsing into immunoglobulin J chain, IgK chain C region and Ig alpha-1 chain C region, which could be directly correlated to the lymphoproliferative process [16]. In addition, we also demonstrated a significant increase of the optical density of the spots related to haptoglobin and alpha-1-antitrypsin, and the presence of nine spots subsequently identified as albumin fragments. It is noteworthy that increased levels of serum haptoglobin and alpha-1-antitrypsin have been already described in patients with several different haematological and non-haematological malignancies, including pancreatic cancers, hepatocellular carcinoma, oral cancers, thyroid carcinomas, and non-small cell lung cancer [17-23]. In particular, an increase of the 21 kDa haptoglobin-related protein (Hpr) (which was the same protein identified in the whole saliva of our patient) has been reported in the serum of patients with malignant lymphoma, with advanced disease and "B" symptoms [23]. Moreover, numerous fragments of albumin and of alpha-1-antitrypsin were detected in the urine of children with cancer [24,25]. To our knowledge, this is the first time that these proteins are described in the whole saliva of a patient with pSS and B-cell NHL. In our case, we also found that Hpr and alpha-1-antitrypsin significantly decreased in response to treatment, suggesting a potential role of these proteins as early therapeutic biomarkers in the clinical setting of lymphoma.

Overall, the patient's salivary profile and its modification over-time represent an indirect proof of the capacity of whole saliva to reflect systemic conditions. Moreover, in this case we demonstrated a specific correspondence between clinical improvement and proteomic changes of the salivary peptide complex, shedding new light on the potential usefulness of proteomic analysis in discovering not only diagnostic but also prognostic and therapeutic biomarkers in patients' whole saliva. We might therefore speculate that, during the follow-up of patients with lymphomas, proteomic analysis might be able to detect salivary biomarkers early predictors of treatment response.

## Conclusions

Overall, once validated in larger studies, these biomarkers could improve patients' management into clinical daily care and facilitate patients' prognostic classification. From the perspective of the research, the analysis of biomarkers signatures in saliva could also help to clarify the pathogenetic pathways underlying lymphoproliferation in pSS leading to develop new concepts for early diagnosis and curative therapies.

## Competing interests

The authors declare that they have no competing interests.

## Authors' contributions

CB, LG and AL designed the study, coordinated the research, analyzed data and wrote the manuscript; SB and LB participated in the design and coordination of study and helped to draft the manuscript; CG carried out statistical analysis; FC, YD and ED carried out proteomic analysis; CB, FF and SG carried out pharmacological treatment; VD carried out the immunohystochemical analysis. All authors read and approved the final manuscript.
